# Multiple Linear Regression Model of Meningococcal Disease in Ukraine: 1992–2015

**DOI:** 10.1155/2020/5105120

**Published:** 2020-02-11

**Authors:** Hennadii Mokhort

**Affiliations:** Department of Epidemiology, Bogomolets National Medical University of the Ministry of Health of Ukraine, Kyiv, Ukraine

## Abstract

Estimating the rates of invasive meningococcal disease (IMD) from epidemiologic data remains critical for making public health decisions. In Ukraine, such estimations have not been performed. We used epidemiological data to develop a national database. These data were used to estimate the population susceptible to IMD and identify the prevalence of asymptomatic carriers of *N. meningitidis* using simple epidemiological models of meningococcal disease that may be used by the national policy makers. The goal was to create simple, easily understood analysis of patterns of the infection within Ukraine that would capture the major features of the infection dynamics. Studies used nationally reported data during 1992–2015. A logic model identified the prevalence of carriage and the proportion of the population susceptible to IMD as key drivers of IMD incidence. Multiple linear regression models for all ages (total population) and for children ≤14 years old were fit to national-level data. Linear models with the incidence of IMD as an outcome were highly associated with carriage and estimated susceptible population in both total population and children (*R*^2^ = 0.994 and *R*^2^ = 0.978, respectively). The susceptibility rate to IMD in the study total population averaged 0.0034 ± 0.0009% annually. At the national level, IMD can be characterized by the simple interaction between the prevalence of asymptomatic carriage and the proportion of the susceptible population. IMD association with prevalence rates of carriage and the proportion of susceptible population is sufficiently strong for national-level planning of intervention strategies for IMD.

## 1. Introduction

The global incidence of invasive meningococcal disease (IMD) ranges from 500,000 to 1,200,000 cases annually; among which 50,000 to 135,000 cases are fatal [1]. Despite the availability of efficient antibiotic therapy and vaccines against various subgroups of meningococcus, meningococcal infection (MI) remains a global health problem. Continued surveillance is needed to predict the dynamic changes in the epidemiology of the disease and to impact the recommendations for current and future vaccines or other prevention strategies [2]. Despite the fact that there are tools for the prevention of IMD, 10–1,000 cases per 100,000 population occur during epidemics in the African meningitis belt, but the incidence of this disease in Europe, North America, and Australia still ranges between 0.3 and 3 cases per 100,000 population [3, 4].

Reduction in morbidity and mortality can be achieved by appropriate and effective control methods and well-developed prevention strategies informed by a high-quality surveillance system. Statistical modeling is an important tool to study the structure of the epidemic process, and multiple regression analysis of empirical data has been successfully applied in countries with different levels of incidence and mortality of IMD to address this issue [5–7]. The incidence of IMD is most commonly used as the dependent variable. The following independent variables (predictors) also have been included: demographic variables (e.g, total population, population density, and percentage of urban population) as well as environmental variables (e.g., percentage of surface by land use or land cover and percentage of water bodies) and atmospheric variables (e.g, wind speed, air temperature, and humidity) [8, 9]. Predictors can also include asymptomatic carriers (presence or density), susceptible population size, herd immunity, smoker prevalence, poverty prevalence, and other indicators of public health [10, 11].

Such analyses have not been conducted in Ukraine, which makes prevention challenging. Our research aimed to integrate epidemiological and microbiological data collected in Ukraine over a 24-year period (1992–2015) to estimate the proportion of the susceptible population and prevalence of asymptomatic meningococcal carriers. Our analyses were developed using the multiple linear regression model, including necessary and most prevalent risk factors [12].

## 2. Materials and Methods

### 2.1. Study Population

Healthy children and adolescents, from orphanages, kindergartens, secondary schools, and colleges, participated in the national disease surveillance system and representative cohorts were selected among all oblasts of Ukraine. During 1992–2015, 954,597 healthy humans (39,775 per year on average) of all ages in Ukraine who had a contact with patients with confirmed MI were tested for meningococcal carriage. In addition, from 1992 to 2015, 508,221 healthy children (21,176 per year on average) were tested for meningococcal carriage in Ukraine with preventive purposes. These were aggregated data without division into age groups. These examinations were performed annually by the Ministry of Health of Ukraine as a part of ordinary monitoring and surveillance standard. Diagnostic tests (or bacteriological studies) were performed by the bacteriological laboratories of the Sanitary Epidemiological Service of Ukraine (currently Public Health Center of the Ministry of Health of Ukraine) including nasopharyngeal swabs transported on culture medium to identify the level of circulating *Neisseria meningitidis* in the healthy population [13]. Bacteriological studies of asymptomatic carriage were carried out in all 26 oblasts of Ukraine.

IMD was defined as a systemic clinical form of meningococcal disease, including meningococcal meningitis, meningococcal septicemia, or mixed forms (meningitis + meningococcemia). Local clinical forms of meningococcal disease (meningococcal nasopharyngitis) were not included in the analysis. In Ukraine, during 1992–2015, 19,940 cases of IMD were registered (831 cases per year on average), including 17,225 cases among children (718 cases per year on average).

Data on the incidence of invasive meningococcal disease were extracted from the reports on particular infectious and parasitic diseases of the Center for Control and Monitoring of Diseases of the Ministry of Health of Ukraine (currently Public Health Center of the Ministry of Health of Ukraine). Indicators of meningococcal carriage were calculated as the percentage of positive results or percentage of infected among the tested in the bacteriological sampling survey on MI as indicator for the Center for Disease Control and Monitoring of the Ministry of Health of Ukraine (currently Public Health Center of the Ministry of Health of Ukraine) (aggregated data in groups of people of all ages (total population) who were in contact with patients with MI and healthy children).

### 2.2. Study Design

We provide a descriptive population-based study of the incidence of invasive meningococcal disease and the carriage of meningococcal infection, which is based on the linear regression model of the epidemic process of meningococcal infection.

### 2.3. Analysis

**Figure 1:**
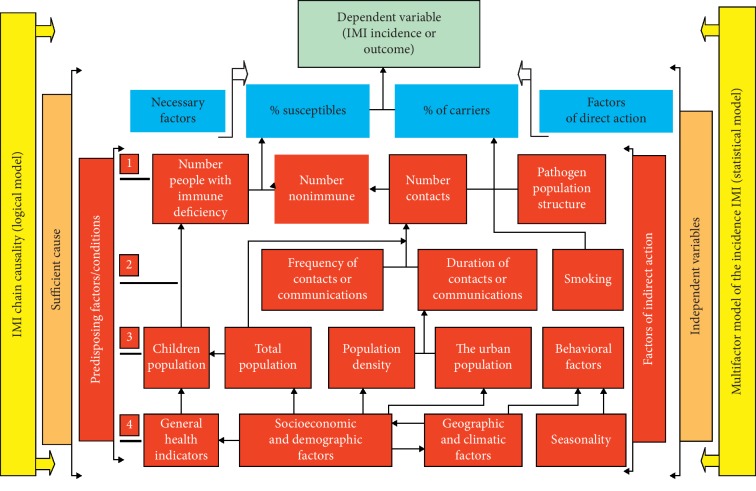
Hierarchical organogram of the mathematical multifactorial model of invasive meningococcal infection (IMI) incidence presenting the independent and dependent variables that lead to an epidemic process. *Legend*: Each cell contains variables or factors that have been used to develop the mathematical model. Connectors indicate the subordination of each cell. It is defined for a given time and place of an epidemic process of meningococcal disease (i.e., invasive meningococcal infection models). The dynamics of invasive meningococcal infection incidence represent a causality chain (logical model) that encompasses all cells, both acting vertically across the system including sufficient causes and independent factors (i.e., predictors or variable). Predisposing factors and factors of indirect action are represented by all red cells. Green horizontal cell = dependent variable = IMI invasive meningococcal infection (IMI incidence or outcome). Yellow vertical cell leftwards (acting vertically across the system) = ІMI chain causality (logical model), including sufficient factors and infection outcome. Golden vertical cell leftwards = sufficient cause. Red vertical cell leftwards = sufficient cause, including predisposing factors and/or conditions and Necessary Factors. Yellow vertical cell on right (acting vertically across the system) = multifactor model of the incidence IMI (statistical model), including independent variables and dependent variable. Golden vertical cell on right = independent variables. Red vertical cell on right = independent factors = factors of indirect action and factors of direct action. Blue horizontal cells = direct risk factors = necessary factors for IMI case to occur, including % of susceptibility (or % of people who became ill when infected) and number of carriers or % carriers. Red vertical cells leftwards and on right = predisposing factors. Red cells first (1) line. Number of people with immune defects: e.g., people with complement deficiency, immunosuppressed. Number of nonimmune healthy people without cellular and humoral immunity against the IMI. Number of contacts: Depending on the duration and frequency of communication between people. Pathogen population structure: The ratio of pathogenic and non-pathogenic agents within a species (capsular and non-capsular meningococcal strains and its relative relationship in laboratory test). Red cells second (2) line. Frequency of contact or communication: number of contacts between people. Duration of contacts or communication: time of intimate contact between people. Smoking: behavioral factor (qualitative). Red cells third (3) line. Children population: Number (or %) of children among the given study population. Total population: Number of people of the study population. Population density: Number of people/km^2^ Urban population: population density in urban settings. Behavioral factors: as national and cultural customs. Red cells fourth (4) line. General health factors: health system accessibility by peoples. Socioeconomic and demographic factors: UNDP human development index. Geography and climatic factors: latitude, landscape, elevation, temperature, humidity, etc. Seasonality: climatic and weather with respect to the study time period.

We developed a logical model of the epidemic process of the infection (see Figure 1). This model is presented as an organogram of hierarchical subordination of various causal factors in the epidemic process of IMI infection[12]. In a general sense, the numbers of IMD depended on the size of the susceptible population and the asymptomatic population, as patients displaying IMD are less likely to transmit the pathogen to the population as a whole. Similarly, we assume that asymptomatic carriers interact randomly with susceptible population members.

The sizes of the susceptible and carrier pools were estimated from retrospective, cross-sectional survey data. The model aggregated the oblast-level data to the national level as the sum of the indicators for all the administrative divisions. The size of the carrier pool was estimated from the prevalence of meningococcal carriage in the survey population. The index of “susceptibles” was derived as the proportion (%) of IMD among meningococcal carriers (approximate proportion of the population susceptible to IMD = APPSIMD) as they developed clinical disease as a result of infection and disease progression. To calculate APPSIMD, we first calculated the annual estimated number of carriers (infected people without clinical manifestations). Then, we calculated the approximate amount of annual meningococcal infected persons as

(1) $$\begin{array}{c} \text{AAQC}=\frac{\text{CPR}\times N\times 365}{D}, \end{array}$$

where AAQC, annual approximate quantity of carriers (infected people without clinical manifestations of IMD); CPR, carrier prevalence rate (the ratio of the carriers detected in the number of examinees); *N,* the number of population of the oblast; 365, days in a year; and *D*, the average duration of carriage (14 days) [14].

The proportion of susceptible population then was calculated as

(2) $$\begin{array}{c} \text{APPSIMD}=\frac{\text{IMD}}{\text{AAQC}}\times 100\%. \end{array}$$

The IMD analysis used multiple regression [15]:

(3) $$\begin{array}{c} Y=a+b_{1}X_{1}+b_{2}X_{2}, \end{array}$$

where *Y,* incidence IMD per 100,000 population; *a, Y* intercept; *X*_1,_ prevalence of carriage (%); *X*_2,_ approximate proportion of susceptible population (APPSIMD); *b*_1_, the regression coefficient for prevalence of carriage; and *b*_2_, the regression coefficient for approximate proportion of the population susceptible.

The predictor and output parameters were tested for normality [16]. Informativeness of the model was characterized by the coefficient of multiple correlation *R*. Mathematical modeling of IMD was carried out in several stages: development of a logical model (see Figure 1), development of a mathematical model, and assessment of the model quality. The main goal of the logical model development was to demonstrate the causal relationship between the various factors involved in the epidemic process of meningococcal disease. The quality of the model was assessed by the indicators of its informativeness, adequacy, stability of Pearson correlation coefficient, and model structure. Calculations were performed using MS Excel 2003 and NCSS 2000 software.

## 3. Results

Thus, the informativeness of models can be considered as sufficient because for 99.4% and 97.8% (coefficients of determination *D* are 0.994 × 100% = 99.4% and 0.978 × 100% = 97.8%) they statistically explain the incidence of invasive meningococcal disease. This indicates high descriptive properties of the model. The forms of both equations had IMD incidence per 100,000 rising with increases in both prevalence of carriage and with the proportion of susceptible populations for total population:

(4) $$\begin{array}{c} Y_{1}=-1.43+0.84X_{1}+455.58X_{2}. \end{array}$$

And for children (0–14 years of age),

(5) $$\begin{array}{c} Y_{2}=-8.58+9.05X_{1}+236.00X_{2}. \end{array}$$

The regression coefficients (*b*_1_ and *b*_2_) and constant (intercept) for IMD were statistically significant (Student exact test: *b*_1_ = 23.15 with *p*=1.97 × 10^−16^*b*_2_ = 26.98 with *p*=8.75 × 10^−18^; *a* = −18.72 with *p*=1.40 × 10^−14^) for the whole population. Similarly, the regression coefficients (*b*_1_ and *b*_2_) and constant (intercept) for IMD were statistically significant (Student exact test: *b*_1_ = 20.66 with *p*=1.95 × 10^−15^; *b*_2_ = 16.02 with *p*=3.02 × 10^−13^; *a* = −10.16 with *p*=1.45 × 10^−9^) for children. Residual analysis of the models did not find any autocorrelation (i.e., almost normal distribution).

The model of the general population is adequate, since the calculated value of *F*-test (895.28) in our case is significantly higher than the *F*-table value (8.75). This model with a 95% probability reflects a set of properties of the epidemic process of meningococcal disease.

The stability of the model or structure of the regression equation corresponds to two basic conditions: (1) the maximum coefficient of pair correlation (*r*) between regressors *X*_1_ and *X*_2_ is less than 0.3–0.5 and is *r*_*X*_1_*X*_2__=0.2959; (2) the coefficients of pair correlation with *Y* in absolute value are much higher than the correlation coefficient between regressors (*r*_*YX*_1__=0.7659 and *r*_*YX*_2__=0.8321 more than *r*_*X*_1_*X*_2__=0.2959). Thus, this model is statistically stable and uncorrelated.

It follows from the linear regression equation of the general population that if the level of meningococcal carriage in Ukraine increases by 1%, the incidence of invasive meningococcal disease increases by 0.84 per 100,000 population. If the susceptible population increases by 1%, then the incidence of IMD increases by 455.58 per 100,000 population.

Overall, there has been a downward trend in the rates of IMD among both total population and children over the course of the study (see Tables 1 and 2). On average, the rates of IMD are about ten times more sensitive to the carriage rate in children (0–14 years of age) than in total population. Ukraine has reduced the incidence of IMD through a decreased number of carriers (*X*_1_) and a decrease of susceptible population percentage (*X*_2_) between 1992 and 2015. We also calculated the approximate annual number of carriers of meningococcal disease, which accounts for one confirmed IMD case (see Table 1) ranging from 17,252 (1992) to 45,967 (2015) with an average of 31,113 carriers.

It is worth noting that this indicator in the general population had a strong tendency towards increase, and among children, it tended to decrease. In our opinion, this difference is due to higher susceptibility of children under the age of 14 to meningococcal infection than the general population, and in the general population, adults who are less susceptible predominate. Thus, a direct benefit of the model is helping assess the efficiency of vaccine prevention of meningococcal disease. The objective criterion for the effectiveness of vaccination is the proportion of susceptible population, which can be calculated by using the parameters of the model. Ultimately, the proportion of susceptible total population occurs within the range of 0.00218% (2015) to 0.0058% (1992) indicating that a limited proportion of the population of Ukraine is susceptible to IMD (see Table 1). Calculations show that an approximate number of people who have been infected with meningococcal disease in Ukraine ranged annually from 13,884,822 to 34,746,741 persons.

## 4. Discussion

The advantage of regression statistical models for epidemiological assessments is the ability to estimate the morbidity and evaluate the effectiveness of vaccine prophylaxis from relatively simple parameters and assumptions. Research on forecasting of meningococcal disease and the efficacy of meningococcal vaccines via regression analysis has been conducted primarily in the United Kingdom and countries of the European Union, North America, New Zealand, and the countries of the meningitis belt [5–7].

In Ukraine, such studies have not been conducted until now. Our regression model is aimed at quantifying the main factors that form the incidence of IMD in Ukraine.

A positive feature of the model is the ability to quantitatively produce a representation (within a given statistical confidence interval) of the complex causal relationships between risk factors and the IMD epidemic process. The latter allows determining the specific weight (importance) of the effect of individual factors on the epidemic process of IMD.

The quality of our models largely depends on the quality of the accessible data. The model used aggregated data from survey values of IMD morbidity and carriage among 26 regions as territorial units. In our paradigm, the susceptible and carrier pools are the only risk factors of IMD emergence and spread in the human population. These variables at least have some potential of being monitored as part of ongoing public health surveillance. All other possible factors that may affect IMD prevalence are acting indirectly through risk of infection (RI) or risk of contamination (number of carriers or percentage of carriers) and risk of susceptibility (RS) or percentage of people who became ill when infected (see Figure 1).

We have made many assumptions and simplifications for this analysis in characterizing the transmission of the pathogen. These include that (1) the level of carriage of meningococcal pathogens does not change within one year; (2) the intensity of the transmission mechanism is relatively stable within one year; (3) the risk of contagion for all members of the population (the total population of Ukraine) is uniform. Such assumptions and simplifications may be acceptable because the size of the susceptible and infected pools is at any given time, a negligible proportion of the entire population.

Our models are further limited by using aggregated passive data from a survey, so that formal residual analysis is limited. It should be stressed that the proposed model does not forecast epidemics, as the quantitative values of the input parameters of the model and the severity of disease for each individual and the period of time are retrospectively identified.

Taking into account the prevalence of asymptomatic meningococcal carriage to construct a regression model of meningococcal disease appears to be one of the most important model variables. Carrier state research can make a significant contribution to our understanding of the epidemiology and pathogenesis of diseases caused by *N. meningitidis* [17].

Another important factor that can negatively affect the quality of our data is the sensitivity of bacteriological tests. The average annual prevalence of meningococcal carriage in Ukraine was reported as 1%. Additionally, in other European countries, 10% of the total population appear to be carriers of *N. meningitidis* [7, 17], suggesting that the prevalence of carriage of meningococcus in Ukraine may be underestimated.

Another important indicator for the construction of the model is the duration of meningococcal carriage. In accordance with our observations of meningococci in carriers with repeated bacteriological examination, the average duration of carriage appears to be 14 days. This value is empirically derived as twice the average incubation period [18]. If the sensitivity of the bacteriological diagnostic of meningococcal infection in Ukraine is less accurate than in other European countries, the duration of the carriage may appear longer than 14 days. Some researchers believe that the duration of meningococcal carriage occurs within the range of 1 to 9 months [6], but other researchers believe that the carriage lasts from several days to months [19].

## 5. Conclusions

The present model can be used as a prototype for the construction of models of those infections that have similar epidemiological patterns (i.e., aerosol transmission, asymptomatic clinical forms, <1% of the population susceptible, and high mortality rate) including pneumococcal, *Haemophilus influenzae* type *b* (Hib) infection, streptococcal, staphylococcal, or diphtheria among others.

The analysis suggests that the nature of MI epidemic process strongly correlates with the prevalence of meningococcal carriage as well as the size/density of susceptible populations, both representing factors of immediate risk of IMD infection and spread. Altogether the present and past surveillance of bacterial meningitis in Ukraine provides a unique source for a comprehensive understanding of the disease dynamics and, most importantly, allows for the development of tools and strategies for disease control and prevention. Thus, our model of the epidemic process of IMD shows a very small and stable proportion of the total population (an average of 0.00343%), which is susceptible to meningococcal infection; therefore, in Ukraine, the change in the incidence of IMD depends mainly on the level of healthy carriage of meningococci.

## Figures and Tables

**Table 1:** Indicators used for building linear regression for total population with invasive meningococcal disease in Ukraine from 1992 to 2015.

Year	IMD incidence, total (*Y*)^*∗*^	Carrier prevalence, (*X*_1_)†	APPSIMD (*X*_2_)^‡^	IMD-predicted rate^§^	AAQC^¶^	Number of carriers/IMD case
1992	2.79	1.856	0.00580	2.63	25,014,717	17,252
1993	2.09	2.353	0.00341	2.78	31,825,516	29,359
1994	2.04	2.241	0.00349	2.10	30,211,677	28,637
1995	2.58	1.992	0.00496	2.04	26,641,182	20,152
1996	2.72	2.620	0.00399	2.51	34,746,741	25,088
1997	2.27	2.420	0.00360	2.61	31,799,375	27,797
1998	2.19	2.172	0.00387	2.25	28,293,959	25,839
1999	2.02	1.935	0.00401	2.16	24,990,644	24,941
2000	1.72	2.335	0.00284	2.01	29,899,981	35,259
2001	1.82	2.139	0.00327	1.82	27,133,125	30,590
2002	1.79	2.098	0.00327	1.85	26,384,436	30,608
2003	1.85	2.290	0.00309	1.81	28,555,695	32,376
2004	2.02	2.006	0.00386	1.90	24,806,647	25,921
2005	1.90	1.923	0.00383	2.01	23,608,051	26,115
2006	1.78	1.493	0.00457	1.92	18,199,540	21,901
2007	1.47	1.590	0.00354	1.88	19,262,233	28,244
2008	1.44	1.387	0.00399	1.49	16,698,407	25,073
2009	1.12	1.731	0.00249	1.52	20,738,600	40,191
2010	1.00	1.468	0.00262	1.12	17,520,208	38,170
2011	1.10	1.926	0.00220	0.95	22,989,542	45,524
2012	0.75	1.301	0.00221	1.16	15,480,263	45,264
2013	0.92	1.236	0.00285	0.90	14,676,973	35,112
2014	0.74	1.177	0.00242	0.66	13,884,822	41,324
2015	0.72	1.275	0.00218	0.63	14,157,912	45,967
Mean	1.70	1.873	0.00343	1.70	24,990,502	31,113
SD^#^	0.62	0.421	0.00090	0.62	5,433,577	8,120

^*∗*^IMD/100,000 total population; ^†^*X*_1_ is expressed as a percentage; ^‡^APPSIMD is expressed as an approximate proportion of the total population susceptible to invasive meningococcal disease, where APPSIMD = (IMD/AAQC) × 100%; ^§^predicted IMD rate/100,000 total population. ^¶^AAQC  = annual approximate quantity of carriers (number of infected people without clinical manifestations of invasive meningococcal disease); ^#^SD = standard deviation.

**Table 2:** Indicators used for building linear regression for 1992–2015 IMD data from children of 0–14 years of age in Ukraine.

Year	Meningococcal disease, incidence, *Y* (age 0–14)^*∗*^	Prevalence of carriage among children, (*X*_1_)^†^	APPSIMD among healthy children aged 0–14, (*X*_2_)^‡^	IMD-predicted rate^§^	AAQC^¶^	Number of carriers/IMD case
1992	11.89	0.98	0.04657	11.29	2,799,994	6,195
1993	11.16	0.77	0.05573	11.53	2,187,512	6,396
1994	10.72	0.99	0.04125	10.18	2,797,353	9,646
1995	10.38	0.73	0.05422	10.86	2,016,011	8,881
1996	11.82	1.26	0.03579	11.23	3,353,170	8,341
1997	10.05	1.18	0.03335	9.96	3,058,046	7,569
1998	11.02	1.34	0.03032	10.69	3,357,296	6,506
1999	9.61	1.15	0.03209	9.43	2,766,959	5,601
2000	7.58	1.25	0.02237	8.00	2,856,649	5,203
2001	8.52	1.36	0.02418	9.44	2,968,831	5,408
2002	8.72	1.44	0.02321	9.94	2,985,671	4,895
2003	9.16	1.07	0.03257	8.82	2,118,231	4,450
2004	10.07	1.12	0.03451	9.70	2,115,410	4,362
2005	9.34	0.99	0.03729	9.24	1,815,270	3,645
2006	9.37	0.92	0.04020	9.27	1,629,424	3,519
2007	8.13	0.74	0.04292	8.29	1,281,344	3,172
2008	8.17	0.72	0.04395	8.36	1,228,660	3,461
2009	6.51	0.72	0.03500	6.16	1,208,599	3,640
2010	5.86	0.62	0.03616	5.58	1,050,988	3,612
2011	6.79	0.80	0.03273	6.35	1,347,199	3,795
2012	4.52	0.82	0.02117	3.82	1,393,352	4,707
2013	5.42	0.50	0,04140	5.74	867,009	9,223
2014	4.10	0.39	0,04000	4.42	687,038	8,179
2015	4.07	0.37	0,04190	4.67	630,662	6,930
Mean	8.46	0.93	0,03660	8.46	2,021,695	5,722
SD^#^	2.39	0.30	0,00940	2.34	880,682	2,038

^*∗*^IMD cases/100,000 children (age 0–14); ^†^*X*_1_ is expressed as a percentage; ^‡^APPSIMD = (IMD/AAQC) × 100%; ^§^predicted IMD rate/100,000 children. ^¶^AAQC = annual approximate quantity of carriers (i.e., # infected children displaying no IMD); ^#^SD = standard deviation.

## Data Availability

The data used to support the findings of this study are included within the article.
